# Long-term reverse remodeling and clinical improvement by MultiPoint Pacing in a randomized, international, Middle Eastern heart failure study

**DOI:** 10.1007/s10840-020-00928-2

**Published:** 2021-06-22

**Authors:** Abdulmohsen Almusaad, Raed Sweidan, Haitham Alanazi, Abdelrahman Jamiel, Fayez Bokhari, Yahya Al Hebaishi, Ahmed Al Fagih, Najib Alrawahi, Amjad Al-Mandalawi, Mohamed Hashim, Bandar Al Ghamdi, Mohammad Amin, Mohamed Elmaghawry, Naeem Al Shoaibi, Antonio Sorgente, Maria Loricchio, Ghaliah AlMohani, Ismail Al Abri, Edmon Benjamin, Nazar Sudan, Alexandre Chami, Nima Badie, Mohammed Sayed, Ahmad Hersi

**Affiliations:** 1 King Abdalaziz Medical City -National Guard Health Affairs, Riyadh, Kingdom of Saudi Arabia; 2 King Fahad Armed Forces Hospital, Jeddah, Kingdom of Saudi Arabia; 3 Prince Sultan Cardiac Center, Riyadh, Kingdom of Saudi Arabia; 4 National Heart Center at the Royal Hospital, Muscat, Oman; 5 Ibn Al-Bitar Cardiac Center, Baghdad, Iraq; 6 Nasiriya Heart Center, Nasiriya, Iraq; 7 King Faisal Hospital and Research Center, Riyadh, Kingdom of Saudi Arabia; 8 Bahrain Defense Force Hospital, Manama, Bahrain; 9 Aswan Heart Centre - Magdi Yacoub Heart Foundation, Aswan, Egypt; 10 King Abdulaziz University Hospital, Jeddah, Kingdom of Saudi Arabia; 11 Cleveland Clinic Abu Dhabi, Abu Dhabi, United Arab Emirates; 12 Sabah Al-Ahmad Cardiac Center, Kuwait City, Kuwait; 13 Abbott, Dubai, United Arab Emirates; 14 Abbott, Sunnyvale, CA USA; 15 King Khalid University Hospital, Riyadh, Kingdom of Saudi Arabia

**Keywords:** Cardiac resynchronization therapy, Heart failure, Reverse remodeling, MultiPoint Pacing

## Abstract

**Purpose:**

Cardiac resynchronization therapy (CRT) with multipoint left ventricular (LV) pacing (MultiPoint™ Pacing, MPP) has been shown to improve CRT response, although MPP response using automated pacing vector programming has not been demonstrated in the Middle East. The purpose of this study was to compare the impact of MPP to conventional biventricular pacing (BiV) using echocardiographic and clinical changes at 6-month post-implant.

**Methods:**

This prospective, randomized study was conducted at 13 Middle Eastern centers. After de novo CRT-D implant (Abbott Unify Quadra MP™ or Quadra Assura MP™) with quadripolar LV lead (Abbott Quartet™), patients were randomized to either BiV or MPP therapy. In BiV patients, the LV pacing vector was selected per standard practice; in MPP patients, the two LV pacing vectors were selected automatically using VectSelect. CRT response was defined at 6-month post-implant by a reduction in LV end-systolic volume (ESV) ≥ 15%.

**Results:**

One hundred and forty-two patients (61 years old, 68% male, NYHA class II/III/IV 19%/75%/6%, 33% ischemic, 57% hypertension, 52% diabetes, 158 ms QRS, 25.8% ejection fraction [EF]) were randomized to either BiV (*N* = 69) or MPP (*N* = 73). After 6 months, MPP vs. BiV patients experienced greater ESV reduction (25.0% vs. 15.3%, *P* = 0.08), greater EF improvement (11.9% vs. 8.6%, *P* = 0.36), significantly greater ESV response rate (68.5% vs. 50.7%, *P* = 0.04), and significantly greater NYHA class improvement rate (80.8% vs. 60.3%, *P* = 0.01).

**Conclusions:**

With MPP and automatic LV vector selection, more CRT patients in the Middle East experienced reverse remodeling and clinical improvement relative to conventional BiV pacing.

## Introduction

Cardiac resynchronization therapy (CRT) provides significant long-term benefits to patients with moderate to severe heart failure (HF), prolonged QRS duration, and reduced ejection fraction (EF) [1–3]. However, up to 40% of patients fail to clinically respond to conventional CRT [4, 5]. Multipoint left ventricular (LV) pacing (MultiPoint™ Pacing [MPP], Abbott, Sylmar, CA), stimulating two LV sites on a quadripolar lead, is one key strategy to improve CRT response over conventional biventricular (BiV) pacing.

Head-to-head comparisons have demonstrated improvements of MPP over BiV in terms of LV pressure response [6–9], LV peak radial strain [10], LV electrical activation [11], and long-term LV function [12–14]. The benefits of the additional LV pacing vector, however, come with the burden of additional programming options. In MPP clinical studies to date, selection of the two LV pacing vectors (each with up to 14 LV cathode-anode combinations) has either (a) been guided by manual, in-clinic electrical or hemodynamic measurements, or (b) left entirely to the discretion of the physician. Consequently, any clinical improvement ultimately observed cannot be directly associated with a single, consistent programming guideline that can be implemented routinely in-clinic. The current study is the first randomized comparison of MPP and BiV in the Middle East that relied solely on one automated, programmer-based tool (VectSelect^TM^, Abbott) which provides MPP LV pacing vector recommendations that are both patient-specific and require minimal physician input.

In this prospective, multicenter investigation, patients were implanted with CRT-D devices and randomized to receive either BiV pacing or MPP. Patient response to CRT was quantified after 6 months by reduction in LV end-systolic volume (ESV) and improvement in LV ejection fraction (EF), both indicative of a reversal of the LV dilation and dysfunction associated with heart failure (i.e., LV reverse remodeling).

## Methods

### Study population

To be enrolled, patients must have been at least 18 years of age, able to provide informed consent, and indicated for de novo CRT-D device implantation. Patients were excluded who did not exhibit left bundle branch block (LBBB), had an epicardial ventricular lead system implanted, exhibited an intrinsic atrial rate below 40 bpm, exhibited atrial fibrillation (AF), had a life expectancy less than 1 year, were pregnant, or dependent on IV inotropic agents. Patients were classified as exhibiting AF if it was either (i) persistent, (ii) permanent and not treated with AV node ablation within 2 weeks of CRT implant, or (iii) documented as paroxysmal or persistent within 30 days of enrollment.

### Study design

This prospective, chronic, randomized, international study was conducted at 13 centers in the Middle East: National Guard Hospital (Riyadh, KSA), King Fahad Armed Forces Hospital (Jeddah, KSA), Prince Sultan Cardiac Center (Riyadh, KSA), Royal Hospital (Muscat, Oman), Ibn Al-Bitar Cardiac Center (Baghdad, Iraq), Nasiriya Heart Center (Nasiriya, Iraq), King Khalid University Hospital (Riyadh, KSA), King Faisal Hospital and Research Center (Riyadh, KSA), Bahrain Defense Force Hospital (Manama, Bahrain), Aswan Heart Centre (Aswan, Egypt), King Abdulaziz University Hospital (Jeddah, KSA), Cleveland Clinic Abu Dhabi (Abu Dhabi, UAE), and Sabah Al-Ahmad Cardiac Center (Kuwait City, Kuwait). All patients provided informed consent, all study protocols were approved by the ethics committee of each institution, and the study was performed in accordance with the ethical standards provided in the 1964 Declaration of Helsinki and its later amendments.

Enrolled patients were implanted with a de novo CRT-D device (Abbott Unify Quadra MP™ or Quadra Assura MP™) and quadripolar LV lead (Abbott Quartet™) according to standard practice. Prior to hospital discharge, patients were randomized into one of two treatment groups, as follows.

1. *BiV Group*: devices programmed to conventional BiV pacing, with the single LV pacing vector selected according to the standard practice of the implanting physician.
2. *MPP Group*: devices programmed with MPP enabled, with the two LV pacing vectors (LV1, LV2) selected using the automatic VectSelect^TM^ feature. Specifically, the VectSelect feature was used to assign LV1 and LV2 cathodes to the LV electrodes with the *earliest* and *latest* RV-LV conduction times (RV-paced to LV-sensed), respectively, with corresponding anodes assigned to yield capture thresholds less than 3.5 V with no phrenic nerve stimulation observed at an output of 1.5x capture threshold.

For both groups, programming of the atrioventricular delay was left to the discretion of the implanting physician. For BiV, the interventricular delay was set to the default value (LV➔RV, 10 ms). For MPP, both the intraventricular and interventricular delays were set to the default values (LV1➔LV2, 5 ms; LV2➔RV, 5 ms).

Echocardiography and 12-lead surface electrocardiography were performed at implant and repeated 6-month post-implant. Echocardiographic metrics were analyzed by blinded core labs. ECG QRS duration (QRSd) was defined as the time from the earliest start time (departure from isoelectric) to the latest end time (return to isoelectric) across all ECG leads, following standard recommendations and ignoring any pre-QRS deflections attributed to pacing artifacts [15].

### Study endpoints

CRT response was characterized by changes in LV end-systolic volume (ΔESV) and LV ejection fraction (ΔEF) at 6-month post-implant. Patients were classified as ESV responders if they demonstrated a relative reduction in ESV of at least 15% vs. baseline. The primary endpoint of the study was a comparison of the proportion of ESV responders (i.e., ESV responder rate) between the BiV and MPP groups. In addition, a combined ESV+EF response classification was also used, in which patients were classified as ESV+EF responders if they demonstrated both a relative reduction in ESV of at least 10% and an absolute improvement in EF of at least 5% vs. baseline [16]. Secondary endpoints included the following comparisons between BiV and MPP groups: ESV reduction, EF improvement, QRS duration (QRSd) reduction, ESV+EF responder rate, and improvement in NYHA functional class.

### Statistical analysis

Statistical analyses were performed using Matlab (Statistics Toolbox, The Mathworks). Categorical variables were reported by patient count and patient percentage, with differences between groups tested using the chi-square test. Differences in responder rates were tested using Fisher’s exact test. Continuous variables were reported as median and interquartile range (IQR), as none demonstrated standard normal distributions according to one-sample Kolmogorov-Smirnov tests. Differences in continuous variables between groups were tested using the Mann-Whitney *U* test. The impact of baseline characteristics on ESV responder rates was quantified using binomial regression. For all statistical tests, *P* < 0.05 was considered statistically significant.

## Results

### Baseline characteristics

One hundred and eighty-two (182) patients were enrolled in 13 centers across 7 Middle Eastern countries. LV lead implantation was unsuccessful in 8 (4.4%) patients, 4 (2.2%) withdrew from the study post-implant, lead dislodgement or system explant occurred in 3 (1.7%), 3 (1.7%) experienced non-cardiac related death, echocardiography was incomplete in 2 (1.1%), and 20 (18.4%) were lost to follow-up. Ultimately, 142 patients contributed complete datasets, with 69 and 73 patients randomized to the BiV and MPP groups, respectively. Baseline patient characteristics were similar for across both groups and are provided in Table 1.

**Table 1:** Baseline characteristics, shown for all patients, biventricular pacing (BiV) patients, and MultiPoint Pacing (MPP) patients

Characteristic	All patients	BiV	MPP	*P* (BiV vs. MPP)
Sample size, *n* (%)	142 (100.0%)	69 (48.6%)	73 (51.4%)	
Male, *n* (%)	96/142 (67.6%)	48/69 (69.6%)	48/73 (65.8%)	0.720
Age, year	61.2 [52.3, 67.9]	59.6 [52.1, 67.0]	62.1 [52.3, 69.4]	0.251
NYHA, *n* (%)				0.186
I	0/142 (0.0%)	0/69 (0.0%)	0/73 (0.0%)	
II	27/142 (19.0%)	16/69 (23.2%)	11/73 (15.1%)	
III	107/142 (75.4%)	50/69 (72.5%)	57/73 (78.1%)	
IV	8/142 (5.6%)	3/69 (4.3%)	5/73 (6.8%)	
Ischemic, *n* (%)	47/142 (33.1%)	21/69 (30.4%)	26/73 (35.6%)	0.593
Hypertension, *n* (%)	81/142 (57.0%)	34/69 (49.3%)	47/73 (64.4%)	0.090
Diabetes, *n* (%)	74/142 (52.1%)	39/69 (56.5%)	35/73 (47.9%)	0.319
QRS duration, ms	158.0 [150.0, 170.0]	158.0 [150.0, 170.0]	160.0 [150.0, 172.3]	0.434
LVESV, mL	135.0 [102.0, 200.0]	140.0 [102.0, 191.0]	130.0 [103.0, 206.0]	1.000
LVEDV, mL	185.5 [148.0, 265.0]	185.0 [163.8, 250.0]	188.0 [145.0, 274.0]	0.920
LVSV, mL	50.0 [39.0, 61.0]	50.0 [40.0, 60.3]	49.0 [37.3, 63.5]	0.612
LVEF, %	25.8 [21.2, 33.3]	25.7 [21.7, 32.5]	26.0 [21.1, 33.6]	0.922
LV lead location [base-apex], *n* (%)				0.121
Basal	29/142 (20.4%)	19/69 (27.5%)	10/73 (13.7%)	
Medial	108/142 (76.1%)	48/69 (69.6%)	61/73 (82.2%)	
Apical	5/142 (3.5%)	2/69 (2.9%)	3/73 (4.1%)	
LV lead location [ant.-post.], n (%)				0.145
Anterior	1/142 (0.7%)	1/69 (1.4%)	0/73 (0.0%)	
Lateral	56/142 (39.4%)	33/69 (47.8%)	23/73 (31.5%)	
Postero-lateral	83/142 (58.5%)	34/69 (49.3%)	49/73 (67.1%)	
Posterior	2/142 (1.4%)	1/69 (1.4%)	1/73 (1.4%)	

*P* values demonstrate differences between BiV and MPP groups

**Fig. 1:**
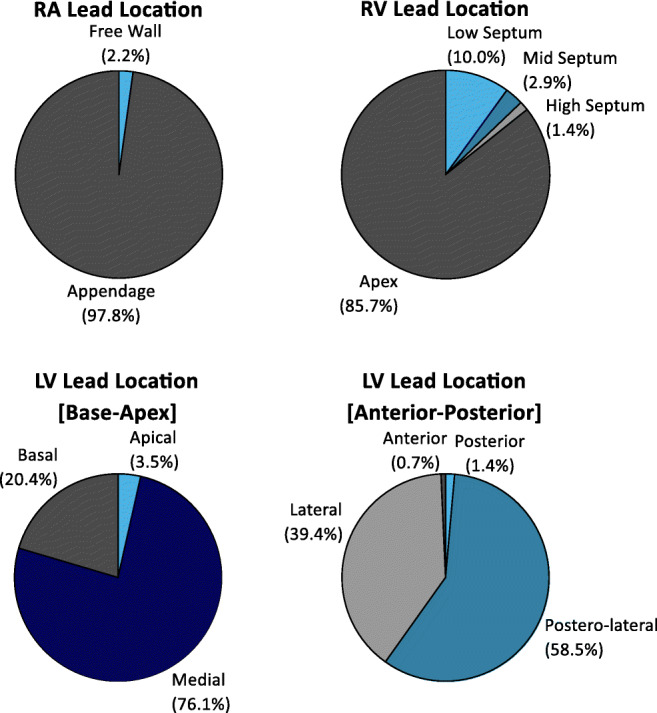
Distribution of implant locations for right atrial (RA), right ventricular (RV), and left ventricular (LV) leads (base-apex, anterior-posterior), as percent of all patients.

The distributions of RA, RV, and LV lead locations are provided in Fig. 1. RA and RV leads were predominantly placed in the RA appendage (97.8%) and RV apex (85.7%), respectively. From base-to-apex, LV leads were predominantly placed medially (76.1%), and from anterior-posterior, LV leads were predominantly placed postero-laterally (58.5%) or laterally (39.4%).

**Fig. 2:**
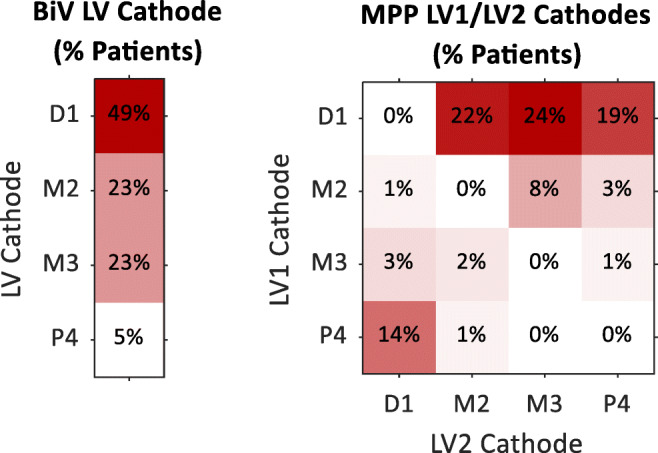
Distribution left ventricular (LV) pacing vector cathodes for biventricular pacing (BiV, left) and MultiPoint Pacing (MPP, right) groups. D1/M2/M3/P4 cathodes correspond to distal tip 1, mid 2, mid 3, proximal 4

The distribution of programmed LV cathodes in the BiV group and programmed LV1 and LV2 cathodes in the MPP group is shown in Fig. 2. The most common LV cathode programmed for BiV patients was D1 (49% of patients). The most common LV1 and LV2 cathodes programmed for MPP patients were D1 (65%) and M3 (32%), respectively. Correspondingly, the most common LV1/LV2 cathode pairs in MPP patients were D1/M3 (24%), D1/M2 (22%), and D1/P4 (19%). The LV1/LV2 cathode pairs were associated with an anatomical separation > 30 mm in 61% of MPP patients.

### CRT response rate

**Fig. 3:**
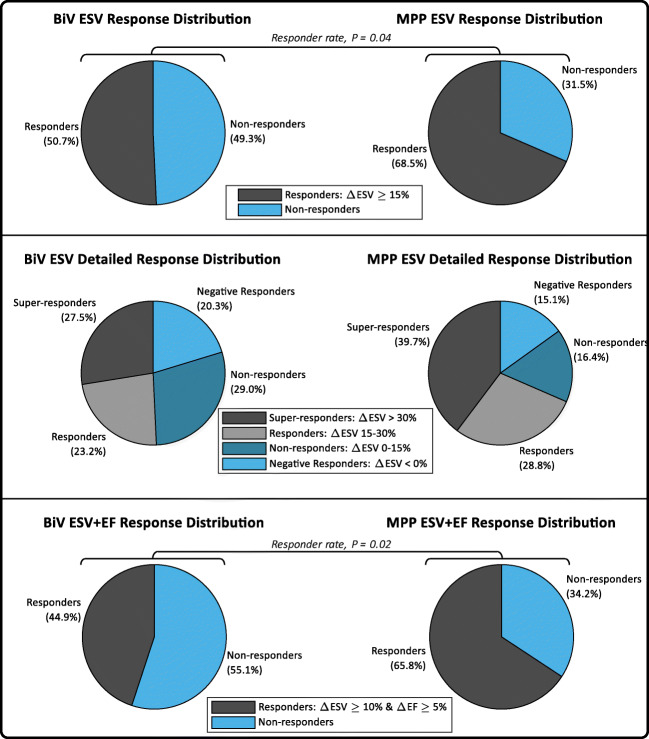
End-systolic volume (ESV) response distribution (top), detailed ESV response distribution (middle), and end-systolic volume + ejection fraction (ESV+EF) response distribution (bottom) for biventricular (BiV) and MultiPoint Pacing (MPP) patients

The MPP group demonstrates higher 6-month CRT responder rates than the BiV group, as shown in Fig. 3. The proportion of patients demonstrating an ESV reduction of 15% or greater was significantly higher in the MPP group (68.5%, 50/73) than in the BiV group (50.7%, 35/69, *P* = 0.04). The proportion of patients demonstrating an ESV reduction of 30% or greater (i.e., “super-responders”) was also higher, but not statistically so, in the MPP group (39.7%, 29/73) than in the BiV group (27.5%, 19/69, *P* = 0.16). In terms of the combined ESV and EF response criteria, the proportion of patients demonstrating an ESV reduction of at least 10% in conjunction with an EF improvement of at least 5% was significantly higher in the MPP group (65.8%, 48/73) than in the BiV group (44.9%, 31/69, *P* = 0.02).

### Reverse remodeling

**Fig. 4:**
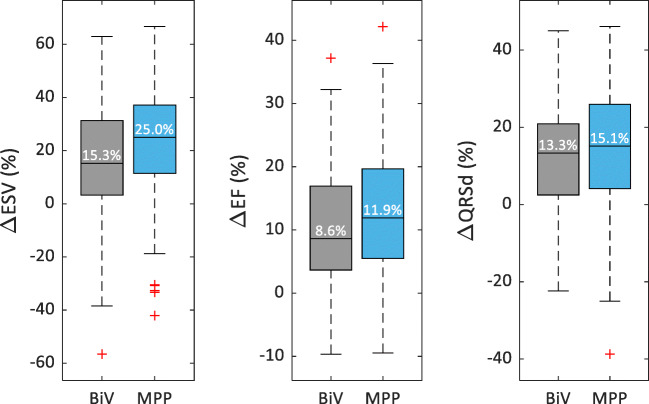
End-systolic volume reduction (ΔESV, left), ejection fraction improvement (ΔEF, center), and QRS duration reduction (ΔQRSd, right) for biventricular (BiV) and MultiPoint Pacing (MPP) patients after 6 months

The LV reverse remodeling effects of CRT are shown in Fig. 4 Patients in the MPP group experienced a greater reduction in ESV (median [IQR] = 25.0% [11.5%, 37.2%]) than patients in the BiV group (15.3% [3.3%, 31.3%], *P* = 0.08). Likewise, patients in the MPP group experienced greater EF improvements (11.9% [5.5%, 19.7%]) than patients in the BiV group (8.6% [3.7%, 16.9%], *P* = 0.36), although statistical significance was not achieved for either metric.

### Electrical synchrony

The impact of CRT on alleviating electrical dyssynchrony can be quantified by changes in QRS duration, also shown in Fig. 4. At 6-month post-implant, patients in the MPP group experienced greater QRS duration narrowing than patients in the BiV group (15.1% [4.1, 26.0] vs. 13.3% [2.5, 20.9], *P* = 0.17).

### NYHA functional class

**Fig. 5:**
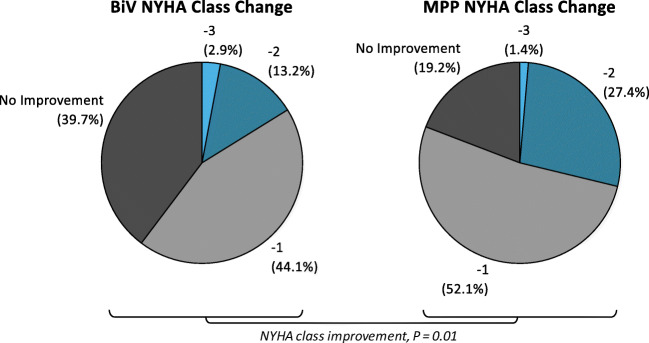
Distribution of changes in NYHA functional class for biventricular (BiV) and MultiPoint Pacing (MPP) patients after 6 months

At 6-month post-implant, significantly more patients in the MPP group experienced an improvement in NYHA functional class (80.8%, 59/73) than those in the BiV group (60.3%, 41/68, *P* = 0.01), as shown in Fig. 5. Furthermore, more patients in the MPP group improved by at least 2 NYHA functional class levels (28.8%, 21/73) than those in the BiV group (16.2%, 11/68, *P* = 0.11), although statistical significance was not achieved for a 2-class improvement.

### Impact of baseline characteristics on response

**Fig. 6:**
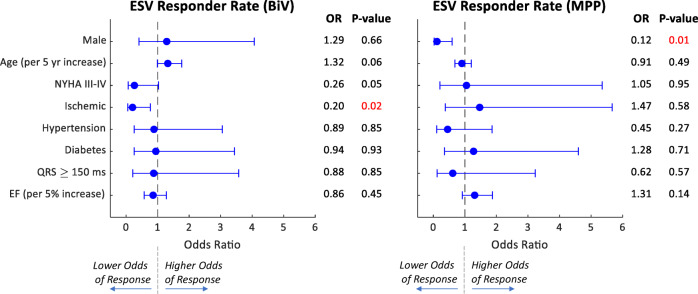
Impact of baseline characteristics on end-systolic volume (ESV) responder rate odds ratio (OR) for biventricular (BiV) and MultiPoint Pacing (MPP) patients, as evaluated by binomial regression

The potential use of baseline characteristics as predictors of ESV response is quantified using binomial regression, with results provided in Fig. 6. For patients with BiV therapy, ischemic cardiomyopathy significantly reduced the odds of ESV response (*P* = 0.02), while age (*P* = 0.06) and poor NYHA class (*P* = 0.05) both approached significance. In contrast, those factors were not predictive of response to MPP. For MPP patients, only gender was a significant predictor (*P* = 0.01). In the MPP group, male patients made up 54.0% of responders but 91.3% of non-responders.

## Discussion

By properly synchronizing both right and left ventricular contraction, CRT can reverse the progressive LV dilation (i.e., remodeling) associated with heart failure, thus improving systolic/diastolic function and, subsequently, clinical outcomes. By adding a second LV pacing site, MultiPoint Pacing has demonstrated promising long-term improvements over conventional biventricular CRT [12–14]. Due to the added programming flexibility of a second LV vector, most MPP devices are left unoptimized with out-of-the-box settings. For CRT implanting centers to achieve the response rates reported by early MPP studies, programming protocols are recommended that often require time-consuming in-clinic measurements.

In the first randomized, multicenter MPP investigation conducted entirely in the Middle East, patients were implanted with CRT-D devices and randomized to receive either BiV or MPP therapy. While LV vector selection in the BiV group was left to the discretion of the implanting physician, the VectSelect^TM^ programmer tool (Abbott) was used to automatically recommend LV vectors for the MPP group, with no external measurements and minimal physician input.

VectSelect recommends MPP LV pacing vectors based on automatic capture threshold and RV-LV conduction time measurements. VectSelect provides two recommendation options for MPP LV cathode pairs: (1) the electrodes with maximal anatomical separation and (2) the earliest and latest activating electrodes. Large-scale clinical trials have previously shown improved response to MPP when LV vectors were selected with > 30 mm anatomical spacing, relative to other MPP configurations [17, 18]. However, the clinical outcomes associated with this strategy were only retrospectively evaluated, leaving it unclear if the vector selection was actually the result of other forms of optimization. Furthermore, the maximal separation option for most leads requires availability of the proximal electrode for > 30 mm separation, which typically has higher capture thresholds. The current study is the first prospective comparison of MPP and BiV that relied solely on the VectSelect programmer tool (Abbott) to automatically recommend MPP LV pacing vectors using the earliest and latest activating electrode option.

Relative to patients receiving conventional BiV therapy, a significantly higher proportion of MPP patients demonstrated an ESV reduction of 15% or more (primary endpoint), and a significantly higher proportion of MPP patients experienced an improvement in NYHA functional class. Changes in ESV, EF, and QRSd all trended toward greater improvement in MPP patients than BiV patients, although statistical significance was not reached in this study for these three metrics. Together, these results point to the enhanced functional and clinical benefits of MPP.

Similar comparisons of MPP and BiV have been reported, but differed in patient population, LV vector selection strategy, and inter-/intraventricular delay programming. In a single-center, 44-patient study, Pappone et al. [12] reported 12-month MPP vs. BiV responder rates for ESV reduction (ΔESV ≥ 15%) of 76% vs. 57%, and for NYHA class improvement of 90% vs. 84%, respectively. While these rates were slightly higher than those observed here (68.5% vs. 50.7% for ESV; 80.8% vs. 60.3% for NYHA), the study by Pappone et al. included LV vector and inter-/intraventricular delay optimization based on comprehensive acute hemodynamic measurements (invasive dP/dt_max_), rather than leveraging automated LV vector selection tools and maintaining nominal (i.e., minimal) inter-/intraventricular delays.

In a retrospective study of 110 CRT patients with optimized LV pacing sites at a single Italian center, Zanon et al. [14] reported higher 12-month MPP vs. BiV responder rates in terms of ESV reduction (90% vs. 72%) and NYHA class improvement (95% vs. 78%). These rates were also slightly higher than this report, even though inter-/intraventricular delays were left at nominal values and not optimized. However, the target LV vein was selected based on manually optimization of either Q-LV time or invasive hemodynamics, with an average of 3 veins tested per patient. In a larger prospective study of 232 patients in 76 Italian centers, Forleo et al. [13] showed 6-month EF improvements with MPP vs. BiV (10.7% vs. 6.5%) that were comparable to those reported here (11.9% vs. 8.6%). Still, MPP LV pacing vector selection was not standardized among centers and included both QRS optimization and maximization of electrical delays between cathodes.

With varied and often time-consuming programming strategies, these research studies were not designed to identify a simple, efficient MPP programming guideline for broad clinical application. Furthermore, all the aforementioned studies were limited to Italian centers, and the results may not be directly applicable worldwide. Not only can the results of the current study be more readily applied to patients in the Middle East, but they may be achievable with minimal physician intervention and without costly or lengthy optimization protocols. Moreover, the reverse modeling response to MPP using VectSelect in this population was not contingent upon commonly cited baseline predictors, such as age, poor NYHA class, long QRSd, low EF, or negative comorbidities (e.g., ischemic cardiomyopathy, hypertension, diabetes), further highlighting its broad application.

The 6-month impact of MPP demonstrated in this study may also point to longer-term benefits. A recent analysis of 436 CRT patients by Rickard et al. [16] evaluated the ability of early echocardiographic changes (9-month echocardiographic follow-up time) to predict longer-term outcomes (5-year clinical follow-up time). Of commonly used reverse left ventricular remodeling metrics (i.e., end-systolic volume, end-diastolic volume, ejection fraction) with various benchmarks, the analysis identified the combined criteria of ESV reduction by 10% and EF improvement by 5% as the most appropriate predictor of patient survival without an LV assist device (LVAD) or heart transplant. According to this response metric, significantly more MPP than BiV patients in this Middle Eastern population (65.8% vs. 44.9% ESV+EF responders) are predicted to survive long-term without requiring more serious intervention.

## Limitations

Although LV vector selection, intraventricular delay, and interventricular delay were all defined a priori for MPP patients, this study left the atrioventricular delay to the physician’s discretion. Consequently, the functional and clinical benefits associated with MPP in this study cannot be attributed to a specific, comprehensive programming strategy. Similarly, for BiV patients, both the atrioventricular delay and LV vector selection were left to the discretion of the implanting physician. A more direct comparison of MPP vs. BiV would require standardized AVD optimization methods and may yield different results.

This study demonstrated the performance of MPP while using automatic vector selection based on cathodal activation times to ease the programming burden. Further studies in this population are needed for a direct comparison to the alternate strategy based on anatomical separation of cathodes.

## Conclusions

In the Middle East, more CRT patients experienced reverse remodeling and clinical improvement with MPP over conventional biventricular pacing when using automatic MPP vector selection.

## Data Availability

Not applicable.
